# Bis(azido-κ*N*^1^)bis­(2,2′-di­pyridyl­amine-κ^2^*N*^1^,*N*^1′^)iron(II) monohydrate

**DOI:** 10.1107/S2414314624011167

**Published:** 2024-11-22

**Authors:** Fatima Setifi, Zouaoui Setifi, Hans Reuter, Mohammad Hadi Al-Douh, Abderezak Addala

**Affiliations:** a Laboratoire de Chimie Ingénierie Moléculaire et Nanostructures (LCIMN) Université Ferhat Abbas Sétif 1 Sétif 19000 Algeria; b Départment de Technologie Faculté de Technologie Université 20 Août 1955-Skikda BP 26 Route d'El-Hadaiek Skikda 21000 Algeria; c Chemistry, Osnabrück University, Barabarstr. 7, 49069 Osnabrück, Germany; d Chemistry Department, Faculty of Science, Hadhramout University, Mukalla, Hadhramout, Yemen; Vienna University of Technology, Austria

**Keywords:** crystal structure, azide, 2,2′-di­pyridyl­amine (dpa), iron(II), hydrogen-bonding

## Abstract

In the hydrated title complex, [Fe(dpa)_2_(N_3_)_2_]·H_2_O (dpa is 2,2′-di­pyridyl­amine, C_10_H_9_N_3_), the Fe^II^ ion is coordinated in a distorted octa­hedral manner by two neutral, chelating dpa ligands and two anionic, monodentate azide (N_3_^−^) ions in a *cis*-configuration. Distortion results from different Fe—N bond lengths [2.1397 (13)–2.2254 (12) Å] and (N—Fe—N)_*cis*_ [80.12 (4)–96.72 (5)°] and (N—Fe—N)_*trans*_ [166.73 (4)–176.62 (5)°] bond angles. Hydrogen bonds exist between two symmetry-related water mol­ecules as hydrogen donors to the γ-N atoms of azido ligands of two adjacent iron complexes and as acceptors from the amide group of the dpa ligands of two additional iron complexes. The hydrogen-bonding pattern results in eight-membered ⋯H—O—H⋯N⋯ rings and a band-like arrangement of the mol­ecules involved. Additional, weaker hydrogen bonds between the α-N atom of the second azido ligand as acceptors and the amide groups of the second dpa ligands as donors cross-link neighboring bands to layers extending parallel to (001).

## Abstract 2

Distortions of the octa­hedral {N_6_}-coordination of the Fe^II^ ion in the title complex Fe(dpa)_2_(N_3_)_2_ are described in terms of bond-length and angle variations alongside to the different kind of inter­molecular hydrogen bonds between the azide ions and the dpa ligands of the complex and the water mol­ecule of crystallization.

## Graphical abstract

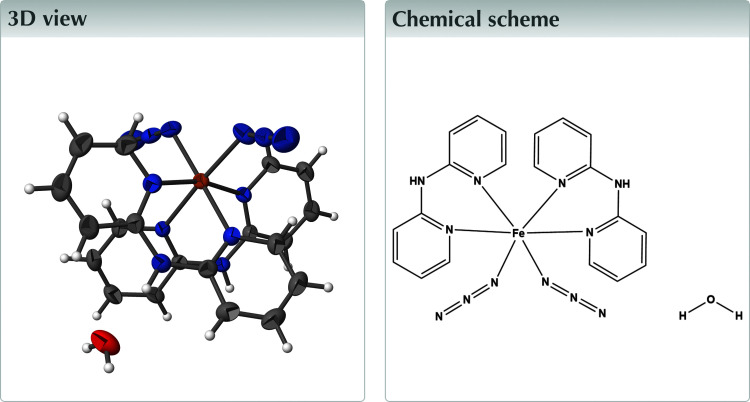

## Structure description

Complexes of first-row transition metals with *d*^4^-, *d*^5^-, *d*^6^- or *d*^7^-configuration can exhibit spin-crossover (SCO) behavior between low-spin and high-spin states in response to external stimuli such as temperature, pressure or light irradiation (Benmansour *et al.*, 2010 ▸). They are of inter­est in functional devices such as sensors, mol­ecular electronics, spintronics, as well as in memory and information processing applications (Halcrow, 2013 ▸). In particular, many electronic devices exploiting the SCO phenomena contain Fe-based SCO materials, which have shown extraordinary performance.

In order to design such SCO materials, our strategy is based on the use of cyano-carbanion ligands. These organic anions are versatile and effective for developing mol­ecular architectures with different topologies and dimensionalities, as a result of their ability to coordinate and bridge metal ions in many different ways (see, for example: Addala *et al.*, 2015 ▸; Cuza *et al.*, 2021 ▸; Dmitrienko *et al.*, 2020 ▸). Continuing our study of SCO 3*d*-metal complexes formed by polydentate and polynitrile units, we describe here the synthesis and crystal structure of the title Fe(II) complex, (I), containing the azido (N_3_^−^) ligand and neutral 2,2′-di­pyridyl­amine, dpa, as co-ligand.

**Figure 1:**
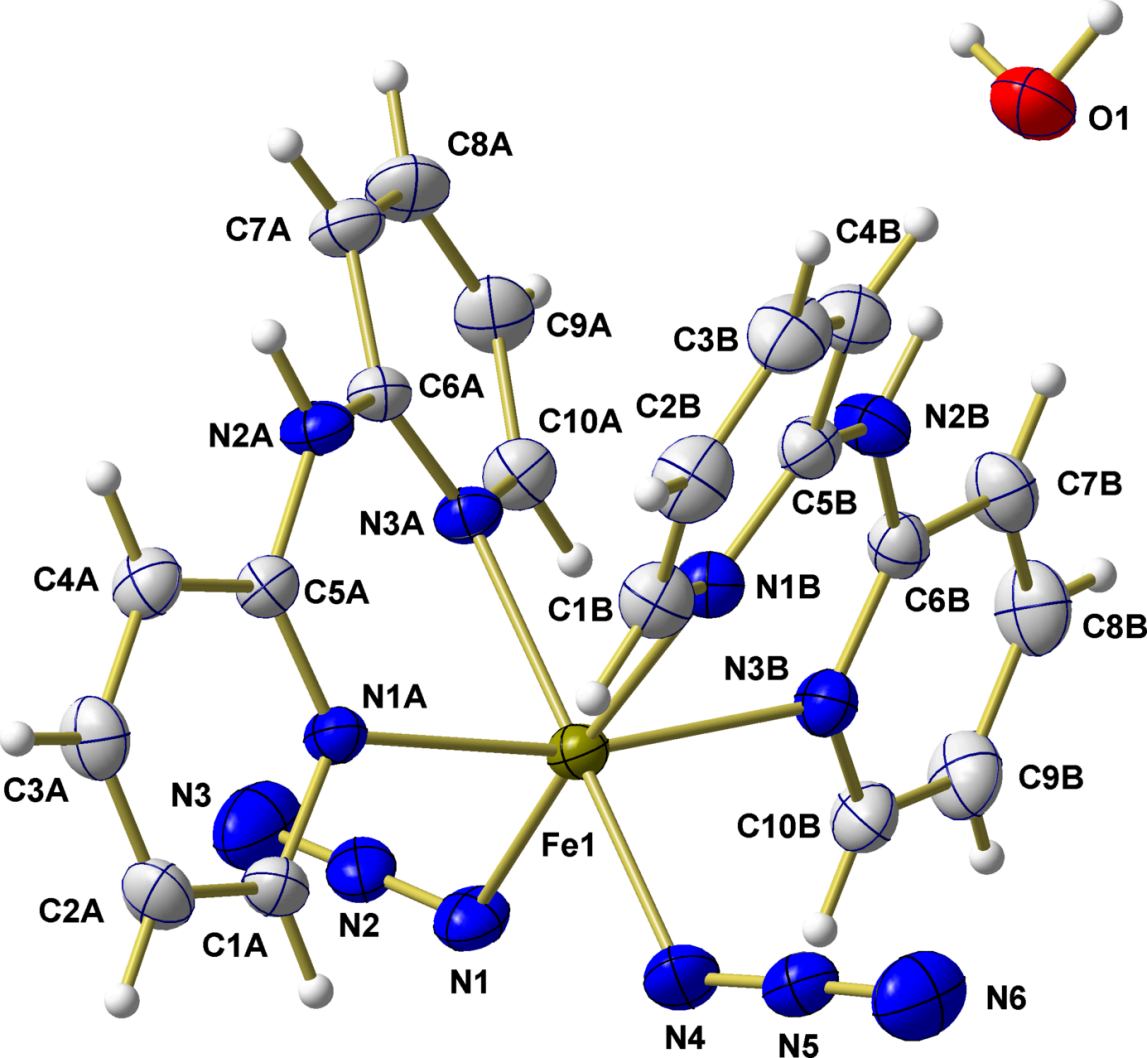
Ball-and-stick model of the asymmetric unit in the crystal structure of the compound Fe^II^(LB_*NN*_)_2_(N_3_)_2_·H_2_O with LB_*NN*_ = dpa showing the atom numbering. With the exception of the hydrogen atoms, which are shown as spheres of arbitrary radius, all other atoms are drawn with displacement ellipsoids at the 40% probability level.

The asymmetric unit of (I) comprises one iron(II) complex and one water mol­ecule (Fig. 1 ▸). The overall composition of the complex corresponds to [Fe^II^(LB_*NN*_)_2_(N_3_)_2_] with two neutral chelating Lewis base (LB) mol­ecules LB_*NN*_ = dpa, and two monodentate azido ligands, N_3_^−^, in a *cis* arrangement.

**Figure 2:**
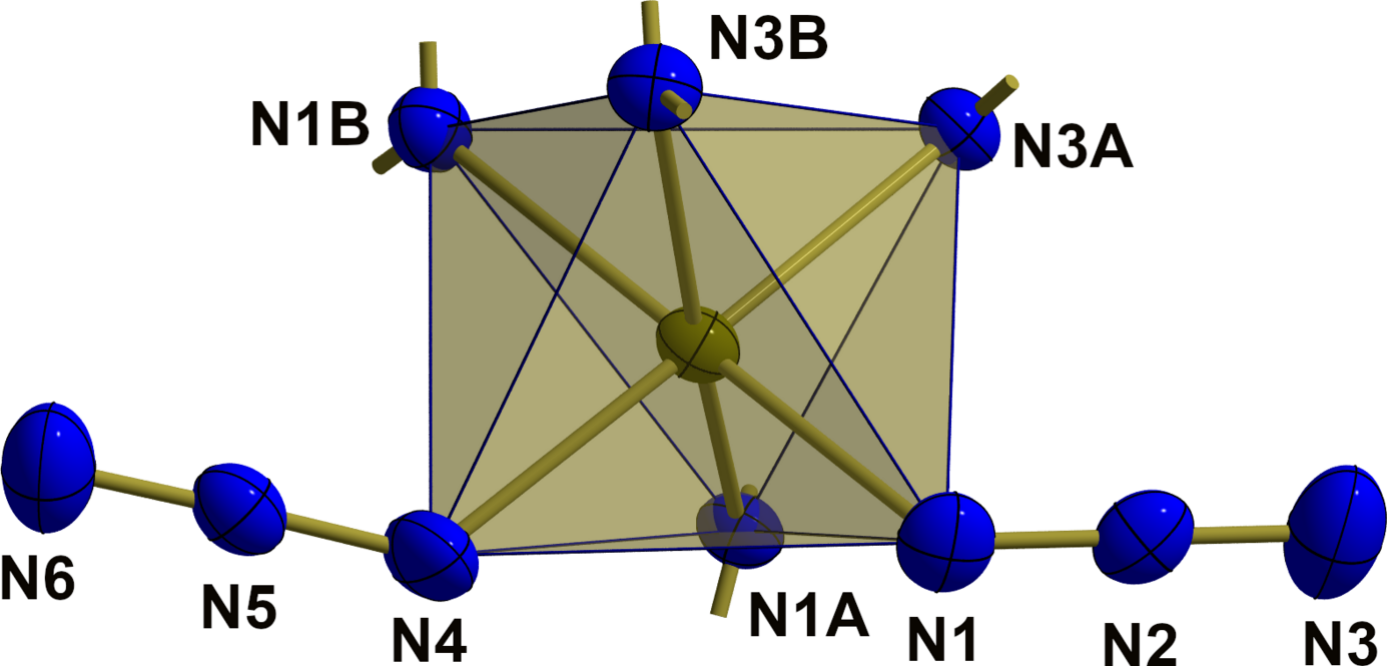
The {FeN_6_} octa­hedron in polyhedral representation, showing the *anti* orientation of both azido ligands. All atoms are drawn with displacement ellipsoids at the 40% probability level. The position of the carbon atoms attached to the nitro­gen atoms of the ligands are indicated as shortened sticks.

**Figure 3:**
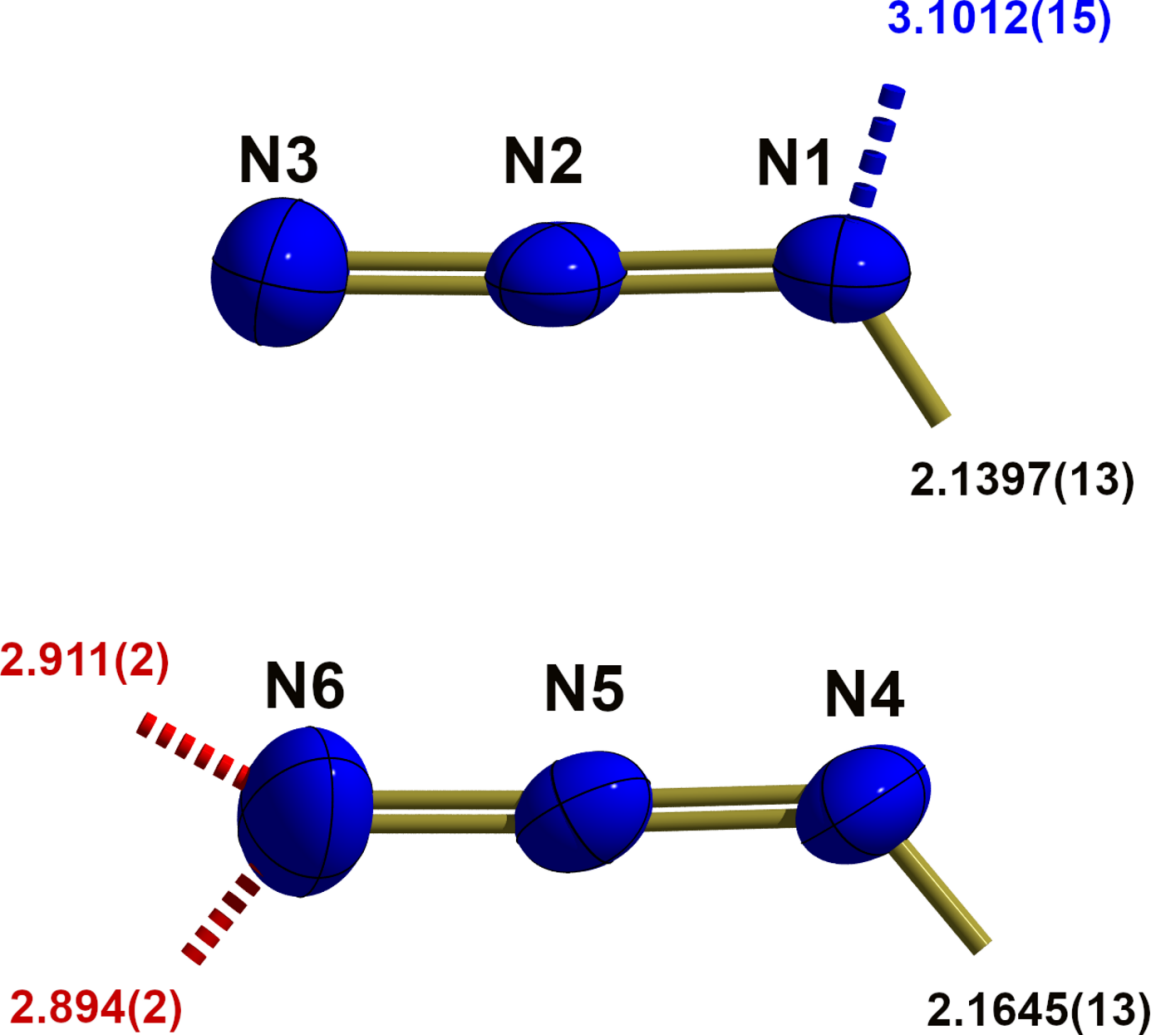
Ball-and-stick models showing the two azido ligands in the iron(II) complex of the title compound in detail, with selected bond lengths [Å], hydrogen bonds [dashed, shortened sticks, d(*D*⋯*A*) in Å, –OH⋯N = red, –NH⋯N = blue) and dative bonds (shortened sticks) to the central iron atom. With the exception of the hydrogen atoms, which are shown as spheres of arbitrary radius, all other atoms are drawn with displacement ellipsoids at the 40% probability level.

The Fe^II^ atom exhibits a slightly distorted octa­hedral {FeN_6_} coordination (Fig. 2 ▸) with an *anti* orientation of the two azido ligands. Distortion results from different Fe—N bond lengths [*d*(Fe—N_azido_) = 2.1397 (13)/2.1645 (13) Å < *d*(Fe—N_dpa_) = 2.1710 (11)–2.2254 (12) Å] and different bond angles [〈(N—Fe—N)_*cis*_ = 80.12 (4)–96.72 (5)°, 〈(N—Fe—N)_*trans*_ = 166.73 (4)–176.62 (5)°]. Both azido ligands are slightly bent with N—N bond lengths corresponding to formal N=N double bonds with the longer one to the metal-coordinating N atom (Table 1 ▸). Moreover, they are different to some extend because of different coordination modes: in the first azido ligand (N1–N3) the iron-coordinating N1 atom is also involved in a hydrogen bond, while in the second azido ligand (N4–N6) the terminal nitro­gen atom N6 is involved in two hydrogen bonds (Fig. 3 ▸).

**Figure 4:**
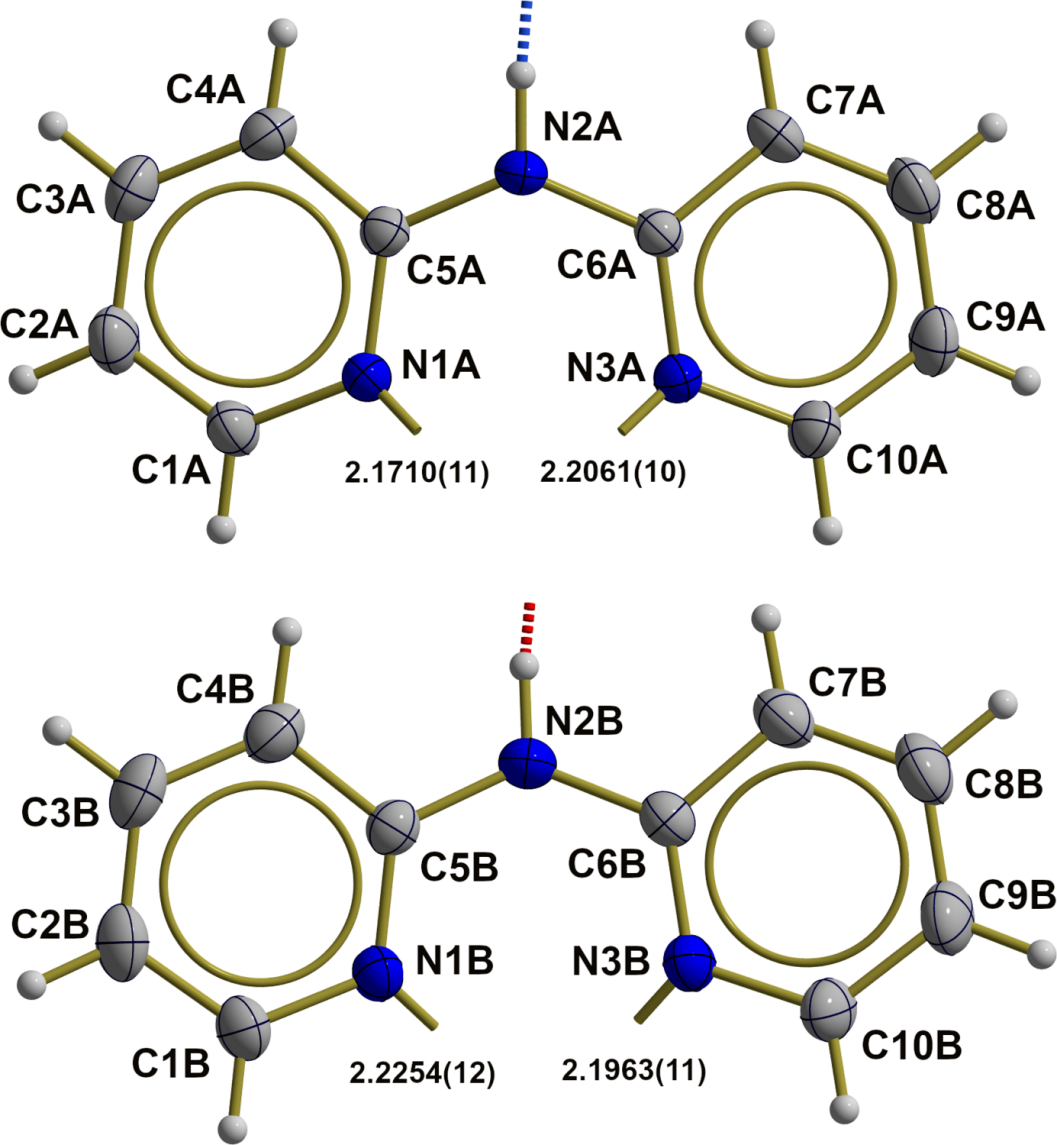
Ball-and-stick models showing the two dpa ligand mol­ecules in the iron(II) complex of the title compound in detail, with selected bond lengths [Å], hydrogen bonds (dashed, shortened sticks, –OH⋯N = red, –NH⋯N = blue) and dative bonds (shortened sticks) to the central iron atom. With the exception of the hydrogen atoms, which are shown as spheres of arbitrary radius, all other atoms are drawn with displacement ellipsoids at the 40% probability level.

The two organic ligands (labeled with suffixes *A* and *B*; Fig. 4 ▸) exhibit very similar conformations characterized by large dihedral angles [24.49 (5)°/17.95 (5)°, *A*/*B*] between the least-squares planes of the two pyridine moieties. N—C and C—C bond lengths and angles are as usual but bond angles at the bridging NH groups are widened [128.64 (10)°/130.43 (12)°, *A*/*B*]. Both amine groups act as hydrogen donors in hydrogen-bonding, N2*A* to the α-N atom of the first azide ion (N1–N3) and N2*B* to the O atom of the water molecule of crystallization. Numerical details of hydrogen-bonding inter­actions of these hydrogen bonds are summarized in Table 2 ▸.

**Figure 5:**
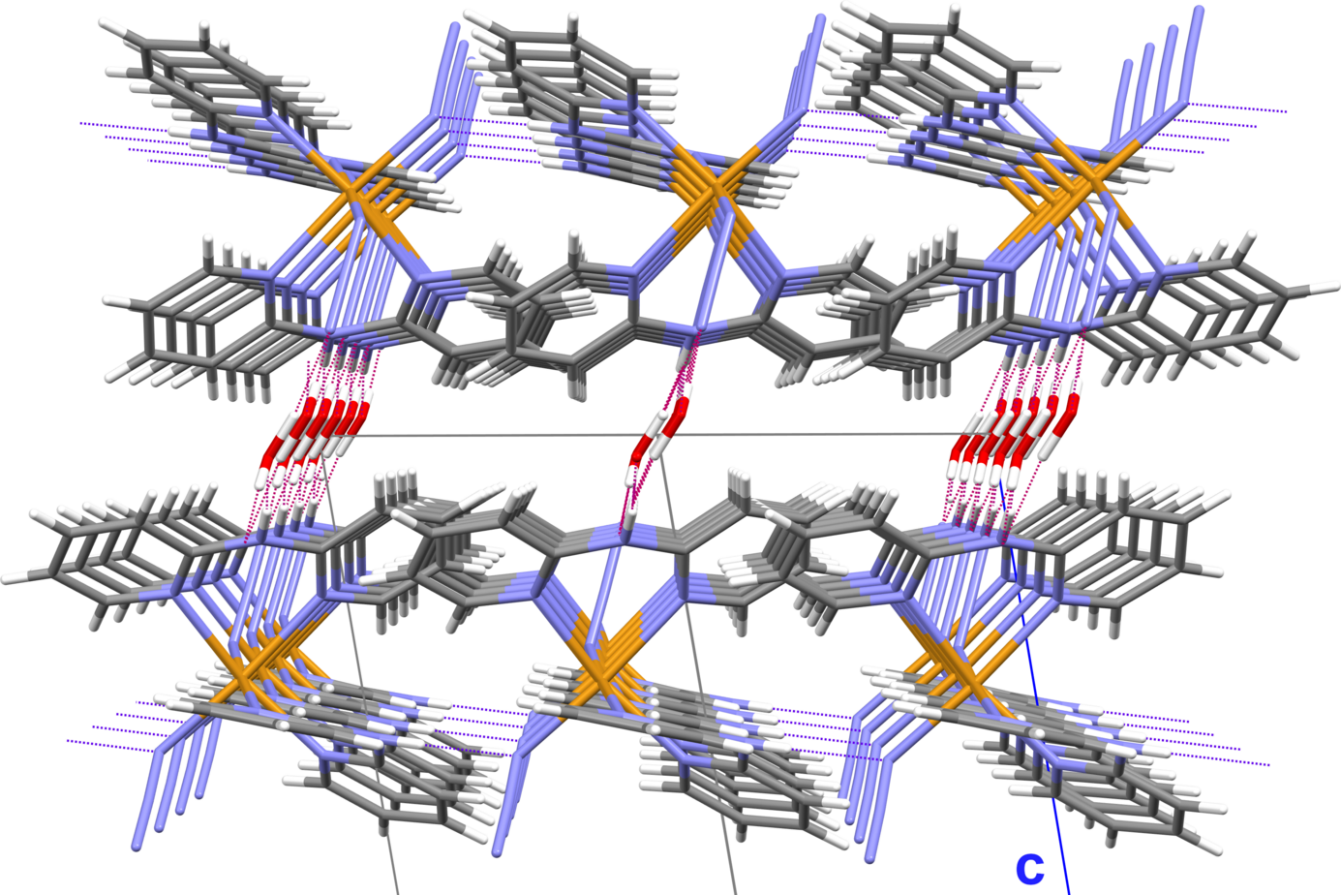
Stick-model showing the crystal packing and hydrogen bonding system in detail in a view along [110]. Atom color code: N = blue, H = white, C = gray, O = red, Fe = bronze. Strong –OH⋯O– and –NH⋯O– hydrogen bonds between the water mol­ecules and the iron complexes responsible for the band-like arrangement of these mol­ecules are visualized as dashed sticks in red, weaker –NH⋯N– hydrogen bonds between the iron complexes of neigboring bands are shown as dashed sticks in blue.

The water mol­ecule acts as hydrogen-bond donor to the γ-nitro­gen atom (N6) of two different azido ligands related to each other *via* a center of symmetry (which also applies to the water mol­ecule) so that an eight-membered ⋯H—O—H⋯N⋯ ring results (Table 2 ▸). Moreover, both water mol­ecules in this ring act as acceptors of additional hydrogen bonds (with H22 from the amide group of the second dpa ligand) of two adjacent iron complexes, which in turn are involved in the formation of further eight-membered rings. In summary, the resulting, supra­molecular system of hydrogen-donor and acceptor bonds between water mol­ecules and iron complexes generates bands expanding parallel to [110]. Weaker hydrogen bonds (Table 2 ▸) between the α-nitro­gen atoms (N1) of the second azido ligand in each iron complex and the hydrogen atoms (H21 of the amide group of the first dpa ligand) cross-link these bands into layers parallel to (001) (Fig. 5 ▸).

Crystal structures of iron(II) complexes of composition Fe^II^(LB_*NN*_)_2_(*X*)_2_ and LB_*NN*_ = dpa have been previously described for *X* = iso­thio­cyanate, NCS (Gaspar *et al.*, 2005 ▸), *X* = dicyanamide, NCNCN (Gaspar *et al.*, 2005 ▸), and *X* = H_2_O with the deca­borate anion [B_10_H_10_]^2−^ as counter-ion (Korolenko *et al.*, 2020 ▸). Other neutral, mononuclear iron(II) complexes of composition Fe^II^(LB_*NN*_)_2_(N_3_)_2_ have been prepared and structurally described in case of LB_*NN*_ = 4-amino-3,5-bis­(2-pyrid­yl)-1,2,4-triazole (Setifi *et al.*, 2021 ▸), LB_*NN*_ = quinolin-8-amine (Setifi *et al.*, 2016 ▸), and LB_*NN*_ = 1,10-phenanthroline (Miao *et al.*, 2006 ▸).

## Synthesis and crystallization

Compound (I) was synthesized under solvothermal conditions from a mixture of iron(II) bis­(tetra­fluorido­borate) hydrate (34 mg, 0.1 mmol), 2,2′-di­pyridyl­amine (34 mg, 0.2 mmol) and sodium azide (13 mg, 0.2 mmol) in a mixture of water and ethanol (4:1 *v*/*v*, 20 ml). This mixture was sealed in a Teflon-lined autoclave and held at 393 K for 2 d, and then cooled to ambient temperature at a rate of 10 K h^−1^ to give the title compound (yield 42%).

## Refinement

Crystal data, data collection and structure refinement details are summarized in Table 3 ▸. The positions of the water H atoms were located from a difference-Fourier map and were refined with a fixed O—H distances of 0.85 Å.

## Supplementary Material

Crystal structure: contains datablock(s) I. DOI: 10.1107/S2414314624011167/wm4222sup1.cif

Structure factors: contains datablock(s) I. DOI: 10.1107/S2414314624011167/wm4222Isup2.hkl

CCDC reference: 2403263

Additional supporting information:  crystallographic information; 3D view; checkCIF report

## Figures and Tables

**Table 1:** Selected geometric parameters (Å, °)

N1—N2	1.1958 (18)	N4—N5	1.1713 (19)
N2—N3	1.153 (2)	N5—N6	1.164 (2)
			
N1—N2—N3	177.99 (16)	N4—N5—N6	178.30 (17)
N2—N1—Fe1	122.09 (9)	N5—N4—Fe1	128.32 (11)

**Table 2:** Hydrogen-bond geometry (Å, °)

*D*—H⋯*A*	*D*—H	H⋯*A*	*D*⋯*A*	*D*—H⋯*A*
N2*A*—H21⋯N1^i^	0.89	2.21	3.1012 (15)	175
N2*B*—H22⋯O1	0.89	1.96	2.8479 (17)	172
O1—H1⋯N6^ii^	0.85 (1)	2.06 (1)	2.894 (2)	167 (3)
O1—H2⋯N6^iii^	0.85 (1)	2.06 (1)	2.911 (2)	176 (3)

Symmetry codes: (i) 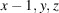; (ii) 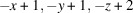; (iii) 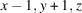.

**Table 3:** Experimental details

Crystal data	
Chemical formula	[Fe(N_3_)_2_(C_10_H_9_N_3_)_2_]·H_2_O
*M*_r_	500.33
Crystal system, space group	Triclinic, *P* 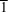
Temperature (K)	300
*a*, *b*, *c* (Å)	7.7496 (5), 9.3778 (6), 16.6178 (10)
α, β, γ (°)	79.516 (3), 83.962 (3), 69.520 (3)
*V* (Å^3^)	1111.34 (12)
*Z*	2
Radiation type	Mo *K*α
μ (mm^−1^)	0.72
Crystal size (mm)	0.38 × 0.21 × 0.12

Data collection	
Diffractometer	Bruker APEXII CCD
Absorption correction	Multi-scan (*SADABS*; Krause *et al.*, 2015 ▸)
No. of measured, independent and observed [*I* > 2σ(*I*)] reflections	93449, 10836, 7610
*R*_int_	0.057
(sin θ/λ)_max_ (Å^−1^)	0.836

Refinement	
*R*[*F*^2^ > 2σ(*F*^2^)], *wR*(*F*^2^), *S*	0.037, 0.109, 1.09
No. of reflections	10836
No. of parameters	315
No. of restraints	2
H-atom treatment	H atoms treated by a mixture of independent and constrained refinement
Δρ_max_, Δρ_min_ (e Å^−3^)	0.38, −0.65

Computer programs: *APEX2* and *SAINT* (Bruker, 2019 ▸), *SHELXT* (Sheldrick 2015*a* ▸), *SHELXL* (Sheldrick, 2015*b* ▸), *DIAMOND* (Brandenburg, 2006 ▸) and *publCIF* (Westrip, 2010 ▸).

## full crystallographic data

### Bis(azido-κN1)bis(2,2'-dipyridylamine-κ2N1,N1')iron(II) monohydrate Crystal data

**Table:**

[Fe(N_3_)_2_(C_10_H_9_N_3_)_2_]·H_2_O	*Z* = 2
*M**_r_* = 500.33	*F*(000) = 516
Triclinic, *P*1	*D*_x_ = 1.495 Mg m^−^^3^
*a* = 7.7496 (5) Å	Mo *K*α radiation, λ = 0.71073 Å
*b* = 9.3778 (6) Å	Cell parameters from 2459 reflections
*c* = 16.6178 (10) Å	θ = 2.7–28.2°
α = 79.516 (3)°	µ = 0.72 mm^−^^1^
β = 83.962 (3)°	*T* = 300 K
γ = 69.520 (3)°	Block, red
*V* = 1111.34 (12) Å^3^	0.38 × 0.21 × 0.12 mm

### Bis(azido-κN1)bis(2,2'-dipyridylamine-κ2N1,N1')iron(II) monohydrate Data collection

**Table:**

Bruker APEXII CCD diffractometer	7610 reflections with *I* > 2σ(*I*)
φ and ω scans	*R*_int_ = 0.057
Absorption correction: multi-scan (SADABS; Krause *et al.*, 2015)	θ_max_ = 36.4°, θ_min_ = 2.4°
	*h* = −12→12
93449 measured reflections	*k* = −15→15
10836 independent reflections	*l* = −27→27

### Bis(azido-κN1)bis(2,2'-dipyridylamine-κ2N1,N1')iron(II) monohydrate Refinement

**Table:**

Refinement on *F*^2^	2 restraints
Least-squares matrix: full	Hydrogen site location: mixed
*R*[*F*^2^ > 2σ(*F*^2^)] = 0.037	H atoms treated by a mixture of independent and constrained refinement
*wR*(*F*^2^) = 0.109	*w* = 1/[σ^2^(*F*_o_^2^) + (0.0356*P*)^2^ + 0.332*P*] where *P* = (*F*_o_^2^ + 2*F*_c_^2^)/3
*S* = 1.09	(Δ/σ)_max_ = 0.001
10836 reflections	Δρ_max_ = 0.38 e Å^−^^3^
315 parameters	Δρ_min_ = −0.65 e Å^−^^3^

### Bis(azido-κN1)bis(2,2'-dipyridylamine-κ2N1,N1')iron(II) monohydrate Special details

**Table:**

Geometry. All esds (except the esd in the dihedral angle between two l.s. planes) are estimated using the full covariance matrix. The cell esds are taken into account individually in the estimation of esds in distances, angles and torsion angles; correlations between esds in cell parameters are only used when they are defined by crystal symmetry. An approximate (isotropic) treatment of cell esds is used for estimating esds involving l.s. planes.
Refinement. The positions of all H atoms were clearly identified in difference-Fourier syntheses. Those of the organic ligands were refined with calculated positions (–CH– = 0.93 Å, –NH– = 0.89 Å) and isotropic displacement parameters depending on the equivalent isotropic temperature factor of the parent atoms. The position of the H atom of the water molecule were refined with fixed O—H distances of 0.85 Å.

### Bis(azido-κN1)bis(2,2'-dipyridylamine-κ2N1,N1')iron(II) monohydrate Fractional atomic coordinates and isotropic or equivalent isotropic displacement parameters (Å^2^)

**Table:**

	*x*	*y*	*z*	*U*_iso_*/*U*_eq_	
Fe1	0.75182 (2)	0.51720 (2)	0.71642 (2)	0.02855 (5)	
N1	0.93226 (16)	0.60191 (16)	0.63027 (8)	0.0407 (3)	
N1A	0.65214 (14)	0.42814 (12)	0.62585 (7)	0.0299 (2)	
N6	1.0198 (2)	0.1417 (2)	0.87772 (11)	0.0623 (4)	
C1A	0.77883 (19)	0.31328 (16)	0.59070 (9)	0.0361 (3)	
H1A	0.902781	0.300924	0.592767	0.043*	
C2A	0.7342 (2)	0.21435 (18)	0.55229 (10)	0.0432 (3)	
H2A	0.825505	0.135541	0.529849	0.052*	
C3A	0.5487 (2)	0.23478 (18)	0.54770 (10)	0.0436 (3)	
H3A	0.514078	0.167702	0.523344	0.052*	
C4A	0.4174 (2)	0.35459 (17)	0.57936 (9)	0.0378 (3)	
H4A	0.292675	0.371479	0.575537	0.045*	
C5A	0.47400 (17)	0.45148 (14)	0.61767 (8)	0.0288 (2)	
N2A	0.33800 (14)	0.57235 (13)	0.64863 (8)	0.0332 (2)	
H21	0.224127	0.575159	0.642404	0.040 (4)*	
C6A	0.34840 (16)	0.71080 (14)	0.66191 (8)	0.0286 (2)	
C7A	0.18479 (19)	0.83846 (16)	0.65752 (10)	0.0388 (3)	
H7A	0.074958	0.829053	0.645695	0.047*	
C8A	0.1891 (2)	0.97676 (17)	0.67087 (11)	0.0458 (3)	
H8A	0.081235	1.061619	0.669914	0.055*	
C9A	0.3551 (2)	0.98975 (16)	0.68581 (10)	0.0426 (3)	
H9A	0.361499	1.083335	0.693806	0.051*	
C10A	0.5092 (2)	0.86086 (15)	0.68847 (9)	0.0363 (3)	
H10A	0.621159	0.870158	0.697129	0.044*	
N3A	0.50850 (14)	0.72022 (12)	0.67923 (7)	0.0298 (2)	
N1B	0.57667 (16)	0.43066 (13)	0.81383 (7)	0.0331 (2)	
C1B	0.5491 (2)	0.29847 (16)	0.80710 (10)	0.0400 (3)	
H1B	0.628281	0.235554	0.771837	0.048*	
C2B	0.4115 (2)	0.25227 (19)	0.84919 (11)	0.0474 (4)	
H2B	0.396359	0.161449	0.841875	0.057*	
C3B	0.2952 (2)	0.3441 (2)	0.90305 (11)	0.0500 (4)	
H3B	0.198122	0.317344	0.931310	0.060*	
C4B	0.3250 (2)	0.4747 (2)	0.91413 (10)	0.0442 (3)	
H4B	0.251129	0.535740	0.951412	0.053*	
C5B	0.46853 (18)	0.51505 (16)	0.86852 (8)	0.0331 (2)	
N2B	0.49131 (17)	0.64924 (14)	0.88067 (8)	0.0389 (3)	
H22	0.403667	0.703840	0.913001	0.044 (5)*	
N2	0.87538 (16)	0.70541 (16)	0.57544 (8)	0.0385 (3)	
C6B	0.64315 (19)	0.69641 (15)	0.86547 (8)	0.0334 (2)	
C7B	0.6399 (2)	0.82180 (18)	0.90159 (10)	0.0451 (3)	
H7B	0.537900	0.870936	0.933549	0.054*	
C8B	0.7890 (3)	0.8704 (2)	0.88898 (12)	0.0530 (4)	
H8B	0.788688	0.954293	0.911503	0.064*	
C9B	0.9408 (3)	0.7933 (2)	0.84230 (11)	0.0519 (4)	
H9B	1.044924	0.822798	0.834127	0.062*	
C10B	0.9336 (2)	0.6727 (2)	0.80852 (10)	0.0423 (3)	
H10B	1.036071	0.620313	0.777860	0.051*	
N3B	0.78511 (16)	0.62598 (14)	0.81743 (7)	0.0347 (2)	
N4	0.98043 (19)	0.30965 (15)	0.75339 (9)	0.0456 (3)	
N3	0.8244 (2)	0.8069 (2)	0.52298 (11)	0.0654 (5)	
N5	0.99782 (16)	0.22607 (14)	0.81594 (8)	0.0388 (3)	
O1	0.2195 (2)	0.85040 (17)	0.97599 (9)	0.0622 (4)	
H1	0.152 (3)	0.838 (3)	1.0184 (9)	0.093*	
H2	0.166 (3)	0.9352 (15)	0.9458 (13)	0.093*	

### Bis(azido-κN1)bis(2,2'-dipyridylamine-κ2N1,N1')iron(II) monohydrate Atomic displacement parameters (Å^2^)

**Table:**

	*U*^11^	*U*^22^	*U*^33^	*U*^12^	*U*^13^	*U*^23^
Fe1	0.02457 (8)	0.02777 (8)	0.03363 (9)	−0.00780 (6)	−0.00253 (6)	−0.00701 (6)
N1	0.0289 (5)	0.0486 (7)	0.0461 (7)	−0.0169 (5)	−0.0018 (5)	−0.0033 (6)
N1A	0.0260 (4)	0.0288 (5)	0.0345 (5)	−0.0065 (4)	−0.0030 (4)	−0.0084 (4)
N6	0.0579 (9)	0.0577 (9)	0.0642 (10)	−0.0215 (8)	−0.0044 (8)	0.0134 (8)
C1A	0.0317 (6)	0.0360 (6)	0.0375 (7)	−0.0050 (5)	−0.0014 (5)	−0.0112 (5)
C2A	0.0472 (8)	0.0376 (7)	0.0430 (8)	−0.0072 (6)	−0.0003 (6)	−0.0170 (6)
C3A	0.0552 (9)	0.0391 (7)	0.0445 (8)	−0.0212 (7)	−0.0039 (7)	−0.0149 (6)
C4A	0.0377 (7)	0.0385 (7)	0.0434 (7)	−0.0181 (6)	−0.0055 (5)	−0.0090 (6)
C5A	0.0287 (5)	0.0266 (5)	0.0317 (6)	−0.0101 (4)	−0.0031 (4)	−0.0035 (4)
N2A	0.0222 (4)	0.0318 (5)	0.0471 (6)	−0.0086 (4)	−0.0024 (4)	−0.0101 (5)
C6A	0.0248 (5)	0.0269 (5)	0.0318 (6)	−0.0061 (4)	−0.0009 (4)	−0.0044 (4)
C7A	0.0269 (6)	0.0348 (6)	0.0493 (8)	−0.0023 (5)	−0.0051 (5)	−0.0076 (6)
C8A	0.0407 (7)	0.0302 (6)	0.0559 (9)	0.0019 (6)	−0.0048 (7)	−0.0071 (6)
C9A	0.0520 (8)	0.0246 (6)	0.0486 (8)	−0.0097 (6)	−0.0039 (7)	−0.0049 (5)
C10A	0.0389 (7)	0.0287 (6)	0.0429 (7)	−0.0133 (5)	−0.0043 (5)	−0.0044 (5)
N3A	0.0260 (4)	0.0253 (4)	0.0378 (5)	−0.0081 (4)	−0.0023 (4)	−0.0052 (4)
N1B	0.0337 (5)	0.0295 (5)	0.0372 (6)	−0.0125 (4)	−0.0005 (4)	−0.0051 (4)
C1B	0.0454 (8)	0.0317 (6)	0.0448 (8)	−0.0155 (6)	−0.0039 (6)	−0.0043 (6)
C2B	0.0535 (9)	0.0408 (8)	0.0542 (9)	−0.0266 (7)	−0.0085 (7)	0.0015 (7)
C3B	0.0449 (8)	0.0574 (10)	0.0523 (9)	−0.0291 (8)	0.0005 (7)	0.0024 (8)
C4B	0.0384 (7)	0.0522 (9)	0.0431 (8)	−0.0189 (7)	0.0048 (6)	−0.0067 (7)
C5B	0.0303 (6)	0.0346 (6)	0.0338 (6)	−0.0109 (5)	−0.0030 (5)	−0.0032 (5)
N2B	0.0360 (6)	0.0383 (6)	0.0448 (7)	−0.0131 (5)	0.0071 (5)	−0.0163 (5)
N2	0.0297 (5)	0.0522 (7)	0.0386 (6)	−0.0203 (5)	0.0003 (4)	−0.0075 (5)
C6B	0.0366 (6)	0.0315 (6)	0.0330 (6)	−0.0111 (5)	−0.0038 (5)	−0.0068 (5)
C7B	0.0543 (9)	0.0404 (7)	0.0453 (8)	−0.0172 (7)	−0.0008 (7)	−0.0165 (6)
C8B	0.0690 (11)	0.0494 (9)	0.0538 (10)	−0.0309 (9)	−0.0064 (8)	−0.0173 (8)
C9B	0.0553 (10)	0.0647 (11)	0.0516 (9)	−0.0363 (9)	−0.0063 (7)	−0.0143 (8)
C10B	0.0368 (7)	0.0543 (9)	0.0427 (8)	−0.0205 (6)	−0.0047 (6)	−0.0130 (7)
N3B	0.0333 (5)	0.0376 (6)	0.0365 (6)	−0.0137 (4)	−0.0039 (4)	−0.0096 (5)
N4	0.0400 (6)	0.0388 (6)	0.0454 (7)	0.0029 (5)	−0.0048 (5)	−0.0058 (5)
N3	0.0534 (9)	0.0789 (12)	0.0559 (9)	−0.0239 (8)	−0.0070 (7)	0.0152 (8)
N5	0.0311 (5)	0.0344 (6)	0.0496 (7)	−0.0085 (4)	−0.0044 (5)	−0.0073 (5)
O1	0.0559 (8)	0.0610 (8)	0.0552 (8)	0.0009 (6)	0.0051 (6)	−0.0191 (7)

### Bis(azido-κN1)bis(2,2'-dipyridylamine-κ2N1,N1')iron(II) monohydrate Geometric parameters (Å, º)

**Table:**

Fe1—N1	2.1397 (13)	C9A—C10A	1.368 (2)
Fe1—N4	2.1645 (13)	C9A—H9A	0.9300
Fe1—N1A	2.1710 (11)	C10A—N3A	1.3572 (16)
Fe1—N3B	2.1963 (11)	C10A—H10A	0.9300
Fe1—N3A	2.2061 (10)	N1B—C5B	1.3386 (17)
Fe1—N1B	2.2254 (12)	N1B—C1B	1.3549 (17)
N1—N2	1.1958 (18)	C1B—C2B	1.367 (2)
N2—N3	1.153 (2)	C1B—H1B	0.9300
N1A—C5A	1.3377 (16)	C2B—C3B	1.387 (3)
N1A—C1A	1.3541 (16)	C2B—H2B	0.9300
N4—N5	1.1713 (19)	C3B—C4B	1.369 (2)
N5—N6	1.164 (2)	C3B—H3B	0.9300
C1A—C2A	1.369 (2)	C4B—C5B	1.403 (2)
C1A—H1A	0.9300	C4B—H4B	0.9300
C2A—C3A	1.391 (2)	C5B—N2B	1.3824 (18)
C2A—H2A	0.9300	N2B—C6B	1.3802 (18)
C3A—C4A	1.370 (2)	N2B—H22	0.8900
C3A—H3A	0.9300	C6B—N3B	1.3350 (18)
C4A—C5A	1.4043 (18)	C6B—C7B	1.4059 (19)
C4A—H4A	0.9300	C7B—C8B	1.367 (2)
C5A—N2A	1.3865 (16)	C7B—H7B	0.9300
N2A—C6A	1.3864 (16)	C8B—C9B	1.387 (3)
N2A—H21	0.8900	C8B—H8B	0.9300
C6A—N3A	1.3383 (15)	C9B—C10B	1.371 (2)
C6A—C7A	1.4042 (17)	C9B—H9B	0.9300
C7A—C8A	1.367 (2)	C10B—N3B	1.3534 (18)
C7A—H7A	0.9300	C10B—H10B	0.9300
C8A—C9A	1.385 (2)	O1—H1	0.850 (1)
C8A—H8A	0.9300	O1—H2	0.850 (1)
			
N1—Fe1—N4	89.86 (5)	N3A—C10A—H10A	118.0
N1—Fe1—N1A	93.92 (5)	C9A—C10A—H10A	118.0
N4—Fe1—N1A	96.72 (5)	C6A—N3A—C10A	117.26 (11)
N1—Fe1—N3B	95.82 (5)	C6A—N3A—Fe1	123.49 (8)
N4—Fe1—N3B	92.31 (5)	C10A—N3A—Fe1	118.17 (9)
N1A—Fe1—N3B	166.73 (4)	C5B—N1B—C1B	117.27 (12)
N1—Fe1—N3A	92.72 (5)	C5B—N1B—Fe1	123.75 (9)
N4—Fe1—N3A	176.62 (5)	C1B—N1B—Fe1	117.56 (10)
N1A—Fe1—N3A	80.94 (4)	N1B—C1B—C2B	123.75 (15)
N3B—Fe1—N3A	89.60 (4)	N1B—C1B—H1B	118.1
N1—Fe1—N1B	175.48 (5)	C2B—C1B—H1B	118.1
N4—Fe1—N1B	88.33 (5)	C1B—C2B—C3B	118.39 (15)
N1A—Fe1—N1B	90.41 (4)	C1B—C2B—H2B	120.8
N3B—Fe1—N1B	80.12 (4)	C3B—C2B—H2B	120.8
N3A—Fe1—N1B	89.25 (4)	C4B—C3B—C2B	119.22 (15)
C5A—N1A—C1A	117.71 (11)	C4B—C3B—H3B	120.4
C5A—N1A—Fe1	123.97 (8)	C2B—C3B—H3B	120.4
C1A—N1A—Fe1	116.25 (8)	C3B—C4B—C5B	119.13 (15)
N1A—C1A—C2A	123.49 (13)	C3B—C4B—H4B	120.4
N1A—C1A—H1A	118.3	C5B—C4B—H4B	120.4
C2A—C1A—H1A	118.3	N1B—C5B—N2B	120.80 (12)
C1A—C2A—C3A	118.27 (13)	N1B—C5B—C4B	122.09 (13)
C1A—C2A—H2A	120.9	N2B—C5B—C4B	117.09 (13)
C3A—C2A—H2A	120.9	C6B—N2B—C5B	130.43 (12)
C4A—C3A—C2A	119.43 (13)	C6B—N2B—H22	113.9
C4A—C3A—H3A	120.3	C5B—N2B—H22	114.1
C2A—C3A—H3A	120.3	N1—N2—N3	177.99 (16)
C3A—C4A—C5A	118.95 (13)	N3B—C6B—N2B	120.69 (12)
C3A—C4A—H4A	120.5	N3B—C6B—C7B	122.07 (13)
C5A—C4A—H4A	120.5	N2B—C6B—C7B	117.23 (13)
N1A—C5A—N2A	120.37 (11)	C8B—C7B—C6B	118.95 (15)
N1A—C5A—C4A	121.97 (12)	C8B—C7B—H7B	120.5
N2A—C5A—C4A	117.64 (11)	C6B—C7B—H7B	120.5
C6A—N2A—C5A	128.64 (10)	C7B—C8B—C9B	119.40 (15)
C6A—N2A—H21	114.1	C7B—C8B—H8B	120.3
C5A—N2A—H21	113.5	C9B—C8B—H8B	120.3
N3A—C6A—N2A	120.69 (10)	C10B—C9B—C8B	118.37 (15)
N3A—C6A—C7A	121.94 (12)	C10B—C9B—H9B	120.8
N2A—C6A—C7A	117.36 (11)	C8B—C9B—H9B	120.8
C8A—C7A—C6A	119.12 (13)	N3B—C10B—C9B	123.44 (15)
C8A—C7A—H7A	120.4	N3B—C10B—H10B	118.3
C6A—C7A—H7A	120.4	C9B—C10B—H10B	118.3
C7A—C8A—C9A	119.60 (13)	C6B—N3B—C10B	117.62 (12)
C7A—C8A—H8A	120.2	C6B—N3B—Fe1	122.47 (9)
C9A—C8A—H8A	120.2	C10B—N3B—Fe1	114.90 (10)
C10A—C9A—C8A	118.04 (13)	N2—N1—Fe1	122.09 (9)
C10A—C9A—H9A	121.0	N4—N5—N6	178.30 (17)
C8A—C9A—H9A	121.0	N5—N4—Fe1	128.32 (11)
N3A—C10A—C9A	123.91 (13)	H1—O1—H2	110 (3)
			
C5A—N1A—C1A—C2A	−4.5 (2)	C5B—N1B—C1B—C2B	3.9 (2)
Fe1—N1A—C1A—C2A	159.85 (13)	Fe1—N1B—C1B—C2B	−163.01 (13)
N1A—C1A—C2A—C3A	1.3 (2)	N1B—C1B—C2B—C3B	−1.2 (3)
C1A—C2A—C3A—C4A	1.8 (2)	C1B—C2B—C3B—C4B	−1.9 (3)
C2A—C3A—C4A—C5A	−1.7 (2)	C2B—C3B—C4B—C5B	2.2 (3)
C1A—N1A—C5A—N2A	−176.65 (12)	C1B—N1B—C5B—N2B	178.21 (13)
Fe1—N1A—C5A—N2A	20.36 (17)	Fe1—N1B—C5B—N2B	−15.71 (18)
C1A—N1A—C5A—C4A	4.54 (19)	C1B—N1B—C5B—C4B	−3.6 (2)
Fe1—N1A—C5A—C4A	−158.45 (11)	Fe1—N1B—C5B—C4B	162.50 (11)
C3A—C4A—C5A—N1A	−1.6 (2)	C3B—C4B—C5B—N1B	0.6 (2)
C3A—C4A—C5A—N2A	179.57 (14)	C3B—C4B—C5B—N2B	178.86 (15)
N1A—C5A—N2A—C6A	26.3 (2)	N1B—C5B—N2B—C6B	−23.0 (2)
C4A—C5A—N2A—C6A	−154.82 (13)	C4B—C5B—N2B—C6B	158.66 (15)
C5A—N2A—C6A—N3A	−29.1 (2)	C5B—N2B—C6B—N3B	15.5 (2)
C5A—N2A—C6A—C7A	151.72 (14)	C5B—N2B—C6B—C7B	−165.32 (15)
N3A—C6A—C7A—C8A	0.4 (2)	N3B—C6B—C7B—C8B	−1.9 (2)
N2A—C6A—C7A—C8A	179.51 (14)	N2B—C6B—C7B—C8B	179.00 (15)
C6A—C7A—C8A—C9A	2.1 (2)	C6B—C7B—C8B—C9B	−1.2 (3)
C7A—C8A—C9A—C10A	−1.5 (2)	C7B—C8B—C9B—C10B	1.7 (3)
C8A—C9A—C10A—N3A	−1.6 (2)	C8B—C9B—C10B—N3B	0.9 (3)
N2A—C6A—N3A—C10A	177.64 (12)	N2B—C6B—N3B—C10B	−176.64 (13)
C7A—C6A—N3A—C10A	−3.25 (19)	C7B—C6B—N3B—C10B	4.3 (2)
N2A—C6A—N3A—Fe1	−14.53 (17)	N2B—C6B—N3B—Fe1	29.71 (18)
C7A—C6A—N3A—Fe1	164.58 (11)	C7B—C6B—N3B—Fe1	−149.40 (12)
C9A—C10A—N3A—C6A	3.9 (2)	C9B—C10B—N3B—C6B	−3.8 (2)
C9A—C10A—N3A—Fe1	−164.56 (12)	C9B—C10B—N3B—Fe1	151.83 (14)

### Bis(azido-κN1)bis(2,2'-dipyridylamine-κ2N1,N1')iron(II) monohydrate Hydrogen-bond geometry (Å, º)

**Table:**

*D*—H···*A*	*D*—H	H···*A*	*D*···*A*	*D*—H···*A*
N2*A*—H21···N1^i^	0.89	2.21	3.1012 (15)	175
N2*B*—H22···O1	0.89	1.96	2.8479 (17)	172
O1—H1···N6^ii^	0.85 (1)	2.06 (1)	2.894 (2)	167 (3)
O1—H2···N6^iii^	0.85 (1)	2.06 (1)	2.911 (2)	176 (3)

Symmetry codes: (i) *x*−1, *y*, *z*; (ii) −*x*+1, −*y*+1, −*z*+2; (iii) *x*−1, *y*+1, *z*.
